# Decision Making from the Experience of Orthognathic Surgery Patients: A Grounded Theory Approach

**DOI:** 10.1177/23800844211014440

**Published:** 2021-05-24

**Authors:** N.R. Paul, S.R. Baker, B.J. Gibson

**Affiliations:** 1 Department of Orthodontics, School of Dental Sciences, Newcastle University, Newcastle upon Tyne, UK; 2 Academic Unit of Oral Health, Dentistry and Society, School of Clinical Dentistry, University of Sheffield, Sheffield, UK

**Keywords:** role of dental professionals, awareness, social support, information, fear, temporality

## Abstract

**Introduction:**

Patients’ decisions to undergo major surgery such as orthognathic treatment are not just about how the decision is made but what influences the decision.

**Objectives:**

The primary objective of the study was to identify the key processes involved in patients’ experience of decision making for orthognathic treatment.

**Methods:**

This study reports some of the findings of a larger grounded theory study. Data were collected through face-to-face interviews of patients who were seen for orthognathic treatment at a teaching hospital in the United Kingdom. Twenty-two participants were recruited (age range 18–66 y), of whom 12 (male = 2, female = 10) were 6 to 8 wk postsurgery, 6 (male = 2, female = 4) were in the decision-making stage, and 4 (male = 0, female = 4) were 1 to 2 y postsurgery. Additional data were also collected from online blogs and forums on jaw surgery. The data analysis stages of grounded theory methodology were undertaken, including open and selective coding.

**Results:**

The study identified the central role of dental care professionals (DCPs) in several underlying processes associated with decision making, including legitimating, mediating, scheduling, projecting, and supporting patients’ decisions. Six categories were related to key aspects of decision making. These were awareness about their underlying dentofacial problems and treatment options available, the information available about the treatment, the temporality of when surgery would be undertaken, the motivations and expectation of patients, social support, and fear of the surgery, hospitalization, and potentially disliking their new face.

**Conclusion:**

The decision-making process for orthognathic treatment is complex, multifactorial, and heavily influenced by the role of DCPs in patient care. Understanding the magnitude of this role will enable DCPs to more clearly participate in improving patients’ decision-making process. The findings of this study can inform future quantitative studies.

**Knowledge Transfer Statement:**

The results of this study can be used both for informing clinical practice around enabling decision making for orthognathic treatment and also for designing future research. The findings can better inform clinicians about the importance of their role in the patients’ decision-making process for orthognathic treatment and the means to improve the patient experience. It is suggested that further research could be conducted to measure some of the key constructs identified within our grounded theory and assess how these change during the treatment process.

## Introduction

The occurrence of dentofacial difference has been reported to be approximately 20% within the general population (Kerawala and Newlands 2009; Wolford and Fields 2000). Orthognathic surgery is offered to patients with dentofacial differences that are beyond the scope of conventional orthodontics (Cunningham and Johal 2015). This elective treatment can have significant consequences for the patient. It involves a lengthy treatment period, although this can vary from country to country. In the United Kingdom, it includes presurgical orthodontics (18–24 mo), orthognathic surgery (typically 2-night hospital stay), and postsurgical orthodontics (up to 12 mo) (Royal College of Surgeons of England 2013). A patient’s decision to undergo orthognathic treatment can, therefore, imply a huge commitment in terms of time, a range of consequences/impacts, and, alongside this, willingness to accept the outcome of treatment in terms of aesthetic and functional changes.

Decision making is not just about how the decision is made but also what influences the decision. In the field of medicine, apart from the medical factors (e.g., the nature, cause, chronicity of the condition), many nonmedical factors, including social context, have been found to influence the decision-making process (McKinlay et al. 1996; Vamos et al. 2009). Characteristics of patients such as their cognitive ability, motivation, perceived social support, and ability to adhere to medical recommendations have been found to influence medical decision making. These factors at times can be more influential than demographics such as, age, sex, ethnicity, or socioeconomic status (Lutfey et al. 2008). The inclusion of patients in the decision-making process has also been found to be important and recommended as part of good-quality care (National Institute of Health and Care Excellence 2019). For example, it has been shown to improve patients’ awareness and acceptance of the final result of orthognathic surgery (Bailey et al. 1999).

At the end of the decision-making process, some individuals have been reported to decline orthognathic surgery, having been offered treatment. Hågensli and colleagues (2014) found that the most common reasons for declining orthognathic surgery were the risks of side effects, the burden of care, and reluctance to undergo surgery. Perhaps linked to this, informed decision making in orthognathic treatment has been found to be problematic, and some patients have reported lacking the necessary information during decision making (Finlay et al. 1995; Stirling et al. 2007; Hågensli et al. 2014). Based on the recommendations of the study by Stirling and colleagues (2007) about the need for better information aids for orthognathic patients, many studies have been undertaken to explore types of information/decision aids for patients, including online information sources (Aldairy et al. 2012), DVDs about orthognathic surgery (Flett et al. 2014), the British Orthodontic Society online information resource (Kettle et al. 2017), orthognathic information clinics (Bergkulla et al. 2017), and orthognathic surgery information on YouTube (Hegarty et al. 2017).

Nevertheless, information provision to patients through information aids only forms one aspect of the patient’s decision-making process (Broder et al. 2000). Broder et al. (2000) explored the “factors” that influence the patient’s decision to undergo orthognathic surgery and concluded that the decision-making process is multifaceted, including interpersonal communication skills (rapport, understanding), resources (social support, financial), and psychosocial factors (stress, motivation). Broder and colleagues (2000) listed all of the factors that influenced patients’ decision making for orthognathic surgery; however, they did not attempt to understand the relationship between these multiple factors or indeed explore how these might be related to important underlying social processes that are important to patients. It could be argued that knowledge about how key factors interrelate or which factors are deemed the most important in terms of the patient experience could lead to a better understanding of a patient’s decision-making process.

The aim of this study was therefore to extend the work of Broder and colleagues (2000) and explore the key processes involved in the experience of decision making in orthognathic treatment. To do this, the study sampled patients at key points in the orthognathic treatment process (presurgery, during treatment, and posttreatment) to gain both prospective and retrospective perspectives on the entire process. The objectives of the study were 1) to identify key processes, according to patients, that play a role in the decision making for orthognathic treatment and 2) identify the type of influence these decision-making processes have on key aspects of the patient’s experience.

## Materials and Methods

Ethical approval was granted for this study from an NHS Research Ethics Committee (ref: 14/LO/1488).

### Design and Approach

This was part of a larger qualitative study using grounded theory (GT) to understand patients’ experience of undergoing orthognathic treatment. The full theory has been reported elsewhere (Paul 2017), and the purpose of the present analysis is to report on patients’ decision-making experience. GT methodology was developed as an interpretive tradition informed by symbolic interactionism (Glaser and Strauss 1967). GT was chosen as the best methodology in the original study to develop a theory based on patients’ experience of orthognathic treatment. It is also a suitable methodology for building theory around the underlying processes involved in patients’ experiences of decision making for orthognathic surgery. This is because GT aims to generate theories that explain social processes or actions through the analysis of data from participants who have experienced them (Sbaraini et al. 2011; Ritchie et al. 2013). This part of the research focuses on decision making and so therefore reports on selectively coded categories around the factors and underlying processes that had influenced patients’ decision making.

### Sampling and Recruitment

**Figure 1.**
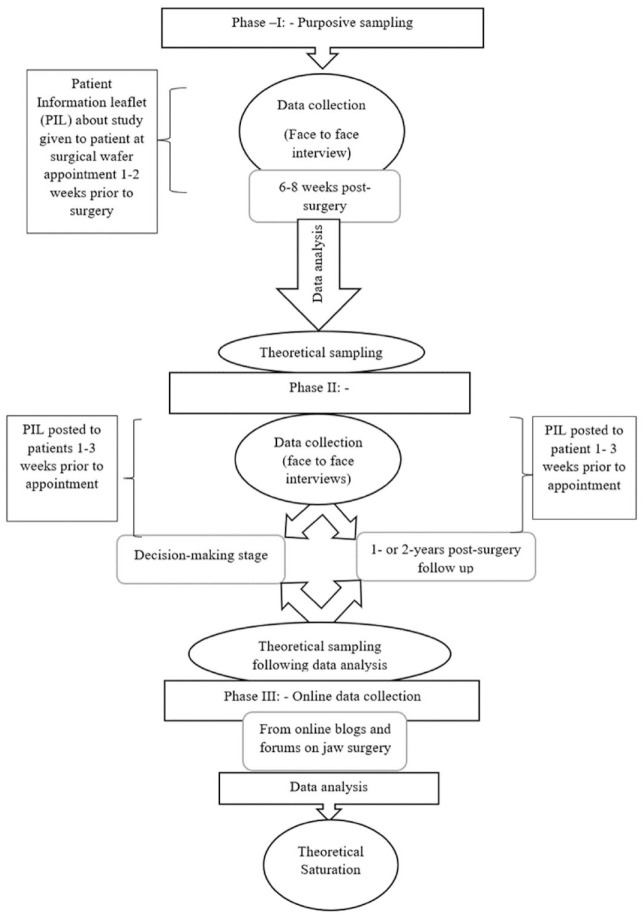
Sampling and recruitment flowchart.

Classic GT focuses on building theory around how the core concerns of people in the area under study are resolved. As a result, sampling and recruitment are undertaken with a view to obtaining a diverse and wide variety of data encompassing differing perspectives on individuals’ concerns in relation to the topic being examined. In this study, participants were sampled initially from a large dental teaching hospital in the United Kingdom (see Figure 1 and Table 1), including those who were 6 to 8 wk postsurgery. Patients were approached with information leaflets about the study when they attended for making a surgical wafer 1 to 2 wk prior to surgery in the oral and maxillofacial surgery (OMFS) clinics. This gave them time to decide if they wished to participate. Participants who wished to take part were formally recruited into the study on the day of discharge from the clinic.

**Table 1.** Sampling and Recruitment of Participants.

Characteristic	Phase 1	Phase 2	Phase 3
Type of sampling	Purposive sampling	Theoretical sampling	Theoretical sampling
Method of data collection	Face-to-face interviews	Face-to-face interviews	Online blogs and forums
Participant’s stage within orthognathic treatment	6-8 wk postsurgery	1. Decision making 2. 1 to 2 y postsurgery	Any stage of orthognathic treatment

Theoretical sampling was also used in this study. Theoretical sampling is defined as “the process of data collection for generating theory whereby the analyst jointly collects, codes and analyses his [*sic*] data and decides what data to collect next and where to find them, in order to develop his [*sic*] theory as it emerges” (Glaser and Strauss 1967, p. 45). The analysis of data obtained from the early interviews with participants 6 to 8 wk postsurgery made it evident that interviewing 2 other groups of patients would help further understanding of the decision-making process. Two groups of participants were subsequently recruited: those in the decision-making stage and those patients in the longer-term follow-up stage after surgery.

In addition to these 3 groups of participants at different stages of the orthognathic journey, online blogs and forums on “jaw surgery” were included to widen the range of data. Forums and blogs written by individuals from around the world were included so as to broaden experiences to outside of the UK context. These online data were selected based on theoretical sampling. That is, they were specifically included to obtain data from people who refused orthognathic treatment and/or who were not satisfied with services provided to them given that these perspectives were missing from our face-to-face interviews. Inclusion of these data led to theoretical saturation, which refers to the constant comparison of conceptual indicators in the data to the point where additional indicators yield no further theoretical specification or elaboration (Holton and Walsh 2017, p. 103).

### Procedure

A topic guide based on the literature review was used for the initial interviews. Most of the interviews were participant led, and open questions such as “Tell me about your experience of making a decision about jaw surgery?” were used to allow participants the freedom to direct the interview to areas that they wanted to talk about. The interviews were voice recorded and transcribed by NRP. The length of the interviews ranged from 20 to 62 min. The data collected from online blogs and forums were copied as quotes and stored in documents that could be analyzed and coded. Blogs and forums on orthognathic/jaw surgery, which did not require any registration, were identified using Google search engine. They were specifically searched to identify blog entries written by patients who were dissatisfied with the outcome of the care provision. Text from such blogs and forums was included in the data analysis to obtain theoretical saturation.

### Research Team and Reflexivity

The research team included 3 members. The researcher who conducted the interviews was the first author (NRP), who carried out the study as part of her PhD in clinical dentistry. NRP is a dentist who was trained in in-depth interviewing and grounded theory for this study. NRP carried out all the data collection and transcribed and coded the data. The second author (BJG) is a professor in medical sociology who has extensive experience as a qualitative researcher. BJG supervised and rechecked the coding and conceptualization of the concepts and factors identified in the study. The third author (SRB) is a professor in health psychology and was the primary supervisor for NRP’s PhD. SRB was responsible for research ethics and the overall conduct of the study. SRB also provided valuable insights into the psychosocial conceptualization within the data.

The researcher (NRP) who carried out the interviews was not part of the participant’s care team and was introduced to participants as a PhD student doing this study. Furthermore, participants were assured that taking part in the study would not affect their care at the hospital in any manner, and anonymity of the participant was ensured by using pseudo-names for each participant. The involvement of BJG and SRB from 2 other disciplines than dentistry enabled better conceptualization of the data, as well as facilitated transparency and trustworthiness of the research findings. With regard to the latter, in the later interviews, the interviewer also carried out member checking (O’Brien et al. 2014) by repeating their understanding of the data to the interviewee for verification during the interview itself. Questions were then asked of participants to further clarify any ambiguities.

### Data Analysis

Data analysis was conducted using the principles of grounded theory (Glaser and Strauss 1967). Initial coding and conceptualization were carried out by NRP and then checked and verified by SRB and BJG. Coding followed the open and selective coding phases of the grounded theory method (Gibson and Hartman 2013). Open coding aims at discovering the main concerns of participants and subsequently to identify the core category, which then enables the researcher to move on to selective coding. Selective coding is the phase in which the researcher focuses on analysis and coding to increasingly specify and develop the theory by identifying its properties and the relationships between categories.

The researcher (NRP) wrote memos about the codes that were developed through the constant comparison of incident to incident within the data (Glaser and Strauss 1967). The written memos were edited and updated continuously through further coding and constant comparison. Finally, the memos were sorted and written up by organizing the categories based on their relationship with each other.

## Results

In this study, a total of 22 participants (aged 18–66 y) were recruited for face-to-face in-depth interviews. The demographics of 3 groups of participants recruited are shown in Table 2. Online blogs and forums were included in this study particularly to obtain data about individuals who decided to not undergo orthognathic surgery since no such data were available from the 22 interviews.

**Table 2.** Demographics of Participants in Face-to-Face Interviews.

Phase	Description	No. of Participants (Male, Female)
1	6–8 wk postsurgery	12 (2, 10)
2	In decision-making phase for surgery	6 (2, 4)
3	1–2 y postsurgery	4 (0, 4)

**Figure 2.**
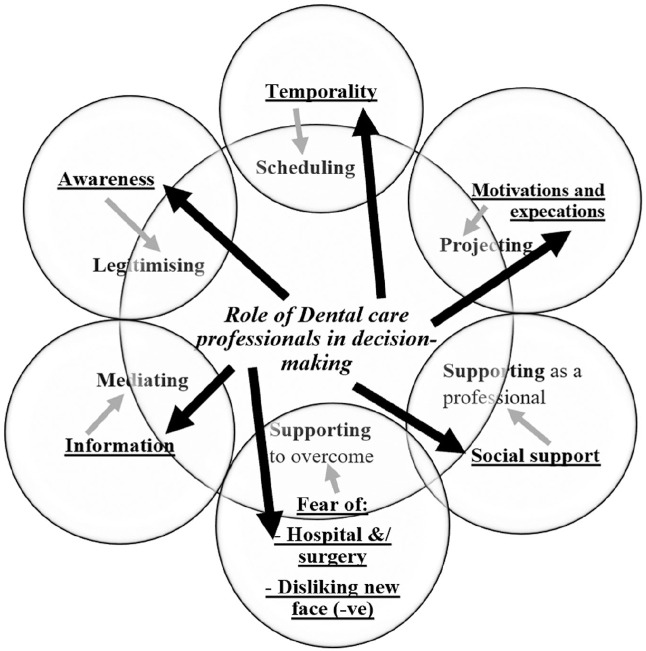
Factors influencing decision making.

The importance of decision making within the patient’s experience of going through orthognathic treatment was evident from reflections made by patients 1 or 2 y postsurgery about their decision. Such reflections often focused on the appropriateness of their decision to undergo orthognathic treatment. The patient’s experience of the decision-making process for orthognathic treatment was found to be influenced by a number of factors and processes (see Fig. 2), each of which will be presented sequentially, briefly discussed, and accompanied by patient quotes to exemplify each category. It is important to note that the role of dental care professionals (dentist, orthodontist, oral and maxillofacial surgeon) was central to several underlying processes that were key to the orthognathic journey, including legitimating, mediating, scheduling, projecting, and supporting decision making. All of these will be highlighted in the following sections.

### Central Role of the Dental Care Professionals in Patients’ Experience of Decision Making for Orthognathic Treatment

Dental care professionals (DCPs) played a central role in patients’ decision making for orthognathic treatment, but this role also extended throughout the care pathway. The role of DCPs was to show understanding and compassion for the patient’s social role and tailor the care pathway accordingly. It was also part of their role to provide professional help in getting through pain, fear, and distress induced by the treatment.

I just put my trust in them [orthodontist and oral surgeon] . . . you know they are the expert. (Lola, 21 y, female, 2 y postsurgery)

They are the expert so I have listened to everything they have said. (Millie, 17 y, female, 6 wk postsurgery)

The patients’ trust in the abilities, experience, and skills of the dental treatment team helped build confidence in their opinion about orthognathic treatment as the best option for them. The approach and support of the orthodontist and their general dentist also played a role in the decision-making process.

My dentist, not within the [name of hospital], my own dentist, she is quite pushy, quite fiery and she was quite pushy about saying well why, why don’t you get it done again, not in a negative way but yeah she seemed quite enthusiastic about me going back and getting it sorted. (Emma, 26 y, female, 8 wk postsurgery)

As seen in the above quote, patients often referred to their general dentist and orthodontist as “my own dentist” and “my own orthodontist,” emphasizing the relationship they had with them. This emphasis on the doctor–patient relationship also highlights the degree of influence these DCPs have on the patients’ decision making.

Data obtained from online blogs and forums also showed the influence of DCPs in the patients’ decision to not undergo orthognathic treatment.

I don’t exactly have an underbite . . . my teeth meet exactly. Looking at my profile, I could benefit from orthognathic surgery (the very suggestion made me put off visiting another orthodontist for 18 years!). But I’m not going with the surgery. . . . The jaw change from surgery would really be very minimal, and I don’t really want an Extreme Makeover. Has anyone else decided “no, thanks . . . surgery’s not for me and I’ll just go with the straight teeth, thanks!” (Valster, online: http://www.archwired.com/phpbb2/viewtopic.php?t=15100)

The above quote is an example of the importance of doctor–patient communication and how some communications can lead to patients’ refusal for orthognathic treatment. The role of doctor–patient communication in decision making has been reported in previous studies. However, the current study identified that the central role of DCPs in patients’ experience of decision making for orthognathic treatment involved engagement with key social processes, including legitimating, mediating, scheduling, projecting, and supporting.

### Awareness and Decision Making in Orthognathic Treatment

When people decide to undergo orthognathic treatment, this often happens because of their increased level of awareness of jaw-related concerns. People can be made aware of their jaw-related concerns through various means, including being victims of name-calling and bullying, photographs (selfies), the influence of peers who have orthodontic treatment, and being told by their dentist. The means by which the patient was made aware of the problem was not reported to heavily affect decision making; it was primarily the psychosocial impact of the awareness that they had that was a key influence on the decision-making process. Patients who were influenced by name-calling, bullying, and teasing, which had left lasting scars on their emotional status, were found to make a decision to undergo orthognathic treatment seemingly without much indecisiveness.

Right it was pointed out to me by a friend who like taking a mickey sometimes at me . . . it was in a joke way but you could tell that there was something wrong with your teeth. So I did [orthognathic surgery] it for myself, like to do the operation and sort it out! . . . Yeah yeah the teasing triggered the decision probably. If it wouldn’t I would have had it myself. I would have known it a bit later that I need straighter teeth and get better looking. (Kenny, 18 y, male, 6 wk postsurgery)

Awareness about available alternative treatment options was also found to be equally important for patients during the decision making for orthognathic treatment.

I found it quite umm clear . . . umm everything was umm well explained, it was very clear what my choices were and it was left to me to make that decision. (April, 19 y, female, 8 wk postsurgery)

All the possible options were given to the patients during their early visits to the hospital to consult the orthodontist and the oral and maxillofacial surgeon. All participants in this study were aware that doing nothing about their concerns was also an option. Understandably, the patients’ awareness of the treatment options was largely gained from the information provided to patients by DCPs who played an important role in *legitimizing* their concerns about their jaw and facial appearance through the clinical diagnosis and treatment plan.

No . . . no when they mentioned that the jaw was out and that I needed some form of surgery that was when I noticed it. . . . They said something about the bite which was a bit daunting because I was there thinking of having a brace, maybe have it for two years. But surgery. . . . (April, 19 y, female, 8 wk postsurgery)

### Information and Decision Making

Patients obtained information regarding treatment options from dental professionals and from various other sources.

I guess I was expecting more comprehensive support throughout this process. So far it seems like I have to ask all the questions, and that no information is volunteered or presented in any way other than verbally. I’m such a novice in this process, and it’s been exhausting to ask question after question and have to dig for information. My mindset alone has shifted on several points since getting serious about braces just over a month ago. (Gabriella36, 33 y, female, online: http://www.archwired.com/phpbb2/viewtopic.php?t=48706)

On the other hand, for some others, too much information was found to be intimidating, especially knowing in detail about what was involved in the surgery.

I felt the less I knew about things the better. . . . Because I think if you overload yourself with information you are always gonna talk yourself out of it. (Lola, 21 y, female, 2 y postsurgery)

Patients who received information with clarity and no perceived coercion were found to benefit more in the decision-making process.

I found it quite clear, everything was umm well explained, it was very clear what my choices were umm and it was left to me to make that decision coz obviously it’s me that has to go, had to go through the treatment. Everyone I spoke to were very clear, yeah I think I found that (decision making) process the simpler part as it were. (Mia, 28 y, female, 8 wk postsurgery)

The DCPs *mediated* the patient’s decision-making process by the provision of trusted information about orthognathic treatment and alternative treatment options, which was found to be an important aspect of an informed decision-making process. All the participants in the semistructured interviews in the study stated that they were well informed about their alternative options, including no treatment as an option.

Various information aids provided by dental professionals to the participants in the current study were a British Orthodontic Society (BOS) patient information leaflet on orthognathic surgery, a DVD by the BOS on orthognathic surgery, and online information about orthognathic surgery, including blogs and support forums. Apart from this, patients were told what to expect during the treatment by the orthodontists and the oral and maxillofacial surgeons treating them. A few patients were also given the opportunity to meet other patients who have had surgery in the hospital in the recent past.

Among the different sources of information, the dental team was the most trusted. While most patients thought that all the information aids were helpful, the leaflets were found to be less useful than other sources. The information provided to the patients was found to be useful for them only if it was relevant to their own needs. The most preferred source of information was firsthand information from a person who had had orthognathic treatment in the past for a concern that was similar to theirs.

I think it’s one of the most useful things being able to talk to people who have gone through it. I think that was what I found most useful. I had one of my friends who had had the surgery, I spoke to her and also I spoke to a lot of people through the blog I wrote online. (Roxy, 22 y, female, 1 y postsurgery)

Most of the younger patients were self-motivated to seek more information online, while others were happy with the information they obtained from the dental care professionals. The online blogs were found to be of good use to many patients when deciding about orthognathic treatment. These blogs and forums provided a wide choice of varied information from which each individual could choose the most appropriate one for them, unlike what the DVD offered.

### Temporality

The concept of temporality (i.e., the time when orthognathic treatment had been offered to the patient in relation to their life events, time given for decision making, and patients’ understanding of time taken for the completion of the treatment) had a strong influence on the decision-making process.

I think it was . . . I had my children and I think there was quite a bit of pain side of it (laughs) got used to the pain side of it, not thinking of any more children now so sorting myself out . . . in a way. (Suzie, female 38 y, 6 wk postsurgery)

The time at which orthognathic treatment is offered to the patient in relation to other important life events played an important role in a patient’s decision about orthognathic treatment. For example, many of the patients undergoing orthognathic surgery were young adults doing their A levels or entering university, and they preferred to defer orthognathic treatment such that it would not interfere with their education and careers. Priorities for people vary at different stages of life, and having orthognathic treatment was one among the many priorities they had. Family and work commitments were the other key life priorities identified in this study, which outweighed orthognathic treatment.

All the participants in the interviews reported that they were given enough time to think and make a decision to undergo orthognathic treatment. However, some patients stated that the time gap between the appointments was a bit too long and so had more time than needed, which, in turn, delayed starting the treatment and completing it.

I think my difficulties in making the decision were really the extent of the treatment needed, the length of time taken and the impact it had on my life especially since I have only a couple of years left in university and then treatment would still be ongoing when I am trying to find a job on the graduate scheme and into obviously that process. (Will, 21 y, male, decision-making phase)

It was found that most patients knew that the treatment would take 2 to 3 y for completion. Therefore, patients were conscious about the decision to schedule orthognathic treatment considering their other life priorities such as career and family commitments. Therefore, these different ideas around the temporality of treatment are directly linked to the role of DCPs. For example, the orthognathic care team determines when orthognathic surgery is scheduled, including considering how this will affect or relate to the patient’s life events. It is in this way that DCPs had a crucial role in *scheduling* the orthognathic surgery for the patient.

### Motivation and Expectations

All the participants in the interviews stated that it was ultimately their own decision to undertake the surgery. This decision was made as a result of internal motivations to improve their appearance or functional abilities. It was not possible to exclude any external influence on the patients’ motivation for orthognathic treatment.

Many patients made reference to “being normal” when they were asked what did they expect from the surgery.

Umm . . . just to look normal. To . . . have a normal smile and a nice smile . . . a normal chin, a normal bottom jaw. . . normal as anyone. (Marie, 66 y, female, 8 wk postsurgery)

This clearly suggested an external influence on the motivation of the patient because this was evidence of a patient’s comparison of oneself with others who were considered normal within society. Therefore, the type of motivation orthognathic patients had when entering treatment appears to be a mix of strong internal motivation marginally influenced by what was considered, by them, to be societal norms with respect to appearance.

Expectations from the surgery were found to be a mixture of functional correction, appearance changes, and psychological improvements.

Leading up to the surgery I was still having speech difficulties, I could speak clearly, annunciating was a problem, I was seeing a speech therapist so I was expecting that would improve. Confidence was the main one really, I just didn’t . . . I would shy away from myself and just didn’t want to draw attention to myself. (Becky, 22 y, female, 2 y postsurgery)

From the interviews in this study, it was found that appearance concerns were the major reason why patients wanted orthognathic treatment. The DCPs had the added burden of managing the expectations of patients, especially since patients expected normality (detailed further in a subsequent article), and what normal actually means is quite a challenging thing to negotiate. Thus, DCPs fulfill an important role in *projecting* the outcome of treatment for patients in terms of its impact on their functional, psychological, and appearance-related well-being.

### Social Support

Social support was drawn from family, friends, and, to some extent, patients’ dental care professionals. The support provided by family and friends in the patient’s decision for orthognathic treatment found in this study was not different from what is reported in the literature already. However, this study also identified the role of dental care professionals in supporting the patient’s decision for orthognathic treatment. DCPs had a central role to play in this process by either *supporting* or resisting these decisions.

Each dentist that I have been to, I have mentioned it but they said it’s extreme to go for the surgery so . . . more or less put me off it and then I moved to [name of town] and then the dentist at [name of another town] suggested it coz he had got people that had it done, sent before for it and it was something he would recommend. So . . . basically swapping dentists made me have it [orthognathic surgery] done. (Suzie, 38 y, female, 8 wk postsurgery)

### Patient Fears—Hospitalization and Negative Results from Surgery

Fear was the single most common reason that negatively affected patients’ decisions to undergo orthognathic surgery. It also might explain why DCPs are so central to the process. Fear of surgery and its consequences, such as pain during recovery and sensory disturbances, was found to be similar to findings in previous studies. However, on exploring patients’ experiences of decision-making processes, their fear of hospitalization, fear of disliking their new face after surgery, and treatment interfering with day-to-day affairs were also found to negatively influence patients’ decision for orthognathic treatment.

Umm having a stay in the hospital is obviously a big negative. . . . Also having the surgery itself in the first place is scary. I have never had surgery before so that also comes in. (Will, 21 y, male, decision-making phase)

When speaking about recovery, most patients were concerned with the pain and swelling during recovery time. Less commonly, patients expressed a fear of weight loss following surgery during the recovery period.

I knew about the weight loss . . . how much will I lose . . . because I wasn’t a big person anyway. Umm even though my husband was doing very high-calorie foods it wasn’t so much how much weight I lost, I probably lost ¾ of a stone but it was the body mass I lost, my clothes were out here (showing action) and umm for about 3–4 months I was like that, you really don’t realise how much weight loss you do with that. That was one of my fears. (Kathy, 52 y, female, 2 y postsurgery)

The last type of fear was the fear of surgery interfering with their daily affairs since orthognathic treatment was an elective procedure. This fear was often projected in patients who were not internally motivated for surgery and had a lower degree of functional and appearance concern. Patients were *supported* by their DCPs to help understand and sometimes overcome these fears through the provision of various forms of information and reassurance often leading to a decision about orthognathic treatment.

## Discussion

This study has identified the central role that DCPs play in patients’ experiences of decision making for orthognathic care. This process was influenced by how DCPs facilitated the interaction between patients and 6 different aspects of the experience, categorized as information awareness, temporality, motivation and expectations, social support, fear of hospitalization, and surgery and disliking their new face. While studies in the past have identified factors associated with decision making for orthognathic treatment (Broder et al. 2000; Stirling et al. 2007), in this article, we have shown the central role that DCPs play in mediating 5 key processes associated with the orthognathic decision-making process. These include legitimating, mediating, scheduling, projecting, and supporting processes. Some of these aspects of the experience have been identified as important factors influencing orthognathic decision making. For example, doctor–patient communication (Broder et al. 2000; Stirling et al. 2007) and trust in the doctor (Boffano et al. 2014) have been shown to be important. Nonetheless, this study for the first time situates the central role DCPs play in mediating key processes associated with patients’ experience of decision making for orthognathic treatment.

The 3 questions addressed in orthognathic decision making according to Broder and colleagues (2000) were “Should I have any treatment?” “Which treatment should I have?” and “When should I have treatment?” It was possible for the patient to answer the first question only if they were aware of their jaw-related concerns and the availability of treatment. While Broder and colleagues (2000) reported that more than 50% of participants in their study had been aware of their jaw-related issue for a long time, the current study also identified the role of awareness of available treatment options in decision making for orthognathic treatment. This leads to the second question, “Which treatment should I have?” (Broder et al. 2000). The answer to this question comes from the information made available to the patients regarding their treatment options. Patients did not favor any particular source of information over another. However, most people expressed benefiting from speaking to somebody of their own age group who had previously had similar surgery. This was congruent with past studies that found that the patient knowing and talking to someone who had completed treatment at their hospital was a significant facilitator for decision making (Cunningham et al. 1996; Broder et al. 2000; Travess et al. 2004; Williams et al. 2005). In addition, this study also found that that there needs to be a balance in how much information is provided to patients about treatment during the decision-making phase. Some people were found to refuse surgery because of the lack of support provided to them by the orthognathic care team. Contrary to Stirling and colleagues’ (2007) finding that orthognathic patients did not make an informed decision about their treatment, the data from this study showed that most patients made informed decisions about orthognathic treatment. Furthermore, what we found was that DCPs play a central role in this.

The category—temporality—identified in this study pertained to the third question, “When should I have treatment?” (Broder et al. 2000). Broder and colleagues (2000) found 15% of orthognathic patients chose to have treatment at that particular time based on their schedule flexibility and financial security. In addition to this, the current study also found that the time at which orthognathic treatment was offered to patients affected their decision because *scheduling* orthognathic treatment has to be carefully considered in relation to other life events and the rank order of each of these. The length of time taken for orthognathic treatment is therefore a central problem to be considered during scheduling, especially since it can clash or affect other life priorities (career, family, and education).

Most patients in this study found orthognathic treatment took too long to complete. This has not been previously considered and indicates that more needs to be done to consider how *scheduling* and in particular the length of the pathway can affect the process. This is possibly because previous studies drew on data from structured questionnaires or interviews that do not allow the patient’s perspective of orthognathic treatment to be explored in qualitative depth. Furthermore, the amount of time given for decision making is another aspect of temporality that was found to influence the patient’s experience of orthognathic treatment. Appropriate time is required for patients to assimilate the information, mobilize resources (financial/personnel), and then make a personal decision. It should be borne in mind, however, that too much time for decision making was also negatively evaluated by some patients who considered this to be a waste of their time. Nonetheless, all participants in the current study stated that sufficient time was given for decision making.

The importance of social support from family and friends in decision making has also been identified in previous research (Holman et al. 1995; Broder et al. 2000; Bhamrah et al. 2015). However, this study has identified the role of DCPs *in supporting* patients by enabling them to make a decision about orthognathic treatment. Motivations and expectations have been studied in the past in relation to assessing patient satisfaction with orthognathic treatment (Nurminen et al. 1999; Chen et al. 2002; Modig et al. 2006; Espeland et al. 2008; Proothi et al. 2010; Oland et al. 2011; Soh and Narayanan 2013). This study, similar to Broder and colleagues (2000), identified the motivations and expectations of patients as a factor that influences their decision to have orthognathic surgery. Concerns over appearance were a major motivating factor for patients to decide to undergo orthognathic treatment, and their expectation for this was to be more “normal,” which demonstrated an internal motivation (patients themselves desired a change in their facial appearance, making them freely opt for orthognathic surgery) with some external influences that made them desire “normality.” However, internally motivated patients have been shown to be more satisfied with the orthognathic treatment process (Cunningham et al. 1996).

Unlike Broder and colleagues (2000), who reported cost, access to care, and cleanliness of facilities as barriers to decision making for orthognathic treatment, the current study found the fear of hospitalization, fear of the surgery, and fear of the negative impacts of orthognathic treatment such as disliking their new face were significant problems for patients. This fear of negative outcomes from treatment, such as the risk of side effects, the burden of care, and a general reluctance to having an operation, has been previously explored in relation to patients’ decisions to have orthognathic treatment (Hågensli et al. 2014; Sadat-Marashi et al. 2015).

Despite these findings, a number of limitations with the study are important to consider. GT methodology provides “analytical generalizations,” not generalizations to a population, and of course this can be limited in scope (Hussein et al. 2014). The current article is reporting on some of the findings of a substantive theory that is considered transferable rather than generalizable to other populations or subjective areas, unlike a formal theory such as status passage (Glaser and Strauss 1971). A further limitation of this study is the sampling. That is, all face-to-face interviews were conducted with patients attending a single surgeon. This may act to reduce the variability observed in the data—which is needed to develop a well-rounded GT. In our study, this limitation was minimized to a certain extent by theoretically sampling data available from online forums and blogs. A further limitation is the gender of participants, who were predominantly female. Given the small proportion of male participants (18%), it may be that certain perspectives on the orthognathic journey were not considered. While there was no evidence that gender played a role in orthognathic decision making, this should not be ruled out on the basis of such a small sample. Further work, including further theoretical sampling, would be required to examine gender-related differences more carefully. With regard to the online data, these were often anonymized, and little demographic information was available. It was therefore not possible to explore age, ethnic, or gender experiences of decision making in the orthognathic treatment process.

In conclusion, the role of DCPs was found to be central in mediating 5 key processes that patients reported were important during their experience of treatment, including legitimating, mediating, scheduling, projecting, and supporting processes. Each of these processes subsequently affected 6 key categories associated with orthognathic care, including information given about treatment and planning, improvements in patients’ awareness of their underlying problem and the potential for this to be treated, how it might be scheduled, how this related to their motivations and expectations, providing social support, and finally enabling patients to overcome their fears of surgery. Future developments in clinical care might consider how these processes and categories related to patient care could be enhanced to enable shared decision making in orthognathic treatment.

## Author Contributions

N.R. Paul, contributed to conception, design, data acquisition, analysis, and interpretation, drafted and critically revised the manuscript; S.R. Baker, contributed to conception, data analysis, and interpretation, critically revised the manuscript; B.J. Gibson, contributed to conception, design, data analysis, and interpretation, critically revised the manuscript. All authors gave final approval and agree to be accountable for all aspects of the work.
